# Structural and Physicochemical Properties of Glycerol-Plasticized Edible Films Made from Pea Protein-Based Emulsions Containing Increasing Concentrations of Candelilla Wax or Oleic Acid

**DOI:** 10.3390/molecules29245998

**Published:** 2024-12-19

**Authors:** Dariusz Kowalczyk, Waldemar Kazimierczak, Emil Zięba, Magdalena Lis, Monika Wawrzkiewicz

**Affiliations:** 1 Department of Biochemistry and Food Chemistry, Faculty of Food Sciences and Biotechnology, University of Life Sciences in Lublin, Skromna 8, 20-704 Lublin, Poland; 2 Department of Biomedicine and Environmental Research, Faculty of Medicine, John Paul II Catholic University of Lublin, Konstantynów 1J, 20-708 Lublin, Poland; waldemar.kazimierczak@kul.pl (W.K.); emil.zieba@kul.pl (E.Z.); magdalena.lis@kul.pl (M.L.); 3 Department of Inorganic Chemistry, Institute of Chemical Sciences, Faculty of Chemistry, Maria Curie-Sklodowska University in Lublin, Maria Curie-Sklodowska Sq. 3, 20-031 Lublin, Poland; monika.wawrzkiewicz@mail.umcs.pl

**Keywords:** pea protein, candelilla wax, oleic acid, emulsion, edible films, microstructure, FTIR, WVP, mechanical properties, heat sealability

## Abstract

Hydrophobization could improve the moisture resistance of biopolymer-based materials, depending on the methods and materials used, providing benefits for packaging applications. The aim of this study was to compare the effect of increasing concentrations (0–2.0%) of candelilla wax (CW) and oleic acid (OA) on the structural and physicochemical properties, including water affinity, of glycerol-plasticized pea protein isolate (PPI) films. OA acidified the film-forming solution and increased its viscosity more effectively than CW. At the highest concentration, OA prevented cohesive film formation, indicating a weakening of protein self-interaction. OA caused less yellowing, matting, and a smaller reduction in UV/VIS light transmittance compared to CW. Both lipids caused a slight reduction in the films’ water content. Phase separation (creaming) of CW enhanced surface hydrophobicity, resulting in a greater reduction in water vapor permeability than OA (~37–63% vs. 2–18%). The addition of lipids did not reduce film solubility or water absorption, and OA even increased these parameters. Increasing lipid content decreased the mechanical strength and stretchability of the films by 28–37% and 18–43%, respectively. The control film exhibited low heat-sealing strength (0.069 N/mm), which improved by 42% and 52% with the addition of CW and OA at optimal levels.

## 1. Introduction

Bioplastics are becoming a key element in the pursuit of a fully sustainable, circular bioeconomy. This is why many countries, including those in the European Union (EU), are actively supporting research and development in this area. Bioplastics represent a diverse group of materials that vary in origin, properties, and disposal methods after use. On an industrial scale, they are produced from both renewable and petrochemical resources. Depending on their origin and biodegradability, they can be classified into three categories:

- Bioplastics derived from renewable resources but not biodegradable, e.g., bio-polyamide and bio-polyethylene;
- Bioplastics derived from fossil (non-renewable) resources that are biodegradable, e.g., polycaprolactone and poly(butylene adipate-co-terephthalate);
- Bioplastics derived from renewable resources that are biodegradable, e.g., starch, cellulose, collagen, and polylactic acid.

Given the need to reduce dependency on depleting fossil resources and address the increasing issue of non-biodegradable waste disposal, the last group of bioplastics is potentially the most eco-friendly [1]. In line with eco-design principles, used bio-packaging should be recyclable, including both aerobic (composting) and anaerobic (biomethanization) organic recycling [2]. It should be noted that if food-grade components such as proteins, polysaccharides, lipids or food additives are used in production, and if manufacturing methods are suitable, the resulting packaging could be consumable along with the packaged product. Edible packaging offers a niche alternative in eco-packaging and can be used wherever typical plastic packaging is restricted. Well-known examples include collagen casings for sausages; capsules made of gelatin, pullulan, starch, or hydroxypropyl methylcellulose; and certain food additives with coating properties applied to fresh fruit (e.g., glycerol esters of wood rosins, sucrose esters of fatty acids, beeswax, candelilla and carnauba wax, and shellac) [3,4].

Approximately 80% of Earth’s living biomass consists of plants [5]. Cultivating plants consumes less energy than animal farming, and plants absorb CO_2_ during growth, contributing to carbon footprint reduction. For example, a meta-analysis showed that, per kilogram of product, fruits and vegetables have a carbon footprint more than 100 times lower than that of ruminant meat. Plant-based biopolymers are therefore potentially more environmentally friendly than animal-based biopolymers [6,7]. Plants can be sustainably cultivated on a very large scale, ensuring a steady supply of raw materials.

Proteins, with their complex structures and versatile functions, stand out as an excellent choice for developing bio-based packaging materials. Unlike polysaccharides, proteins offer a unique combination of properties that can be fine-tuned based on their origin and amino acid profile. When selecting plant-based proteins for packaging production, it is important to consider their key properties, such as the ability to form effective barriers, mechanical durability, optical properties, processability, and compatibility with various production methods, as well as economic and legal factors like availability, cost, and regulatory compliance [8].

Pea protein isolate (PPI) is regarded as a high-quality, functional ingredient in the food industry because of its low allergenic potential, high protein content, widespread production ability, affordability, and origin from a sustainable and non-genetically modified crop. Additionally, pea protein exhibits relatively good functional properties, including solubility, water and oil retention, emulsifying ability, gel formation, and viscosity. These characteristics make pea protein a highly promising ingredient in the food industry [9], including production edible packaging [10].

Some protein-based films demonstrate excellent barriers to oxygen, aroma, and lipids at low relative humidity (RH). However, due to their hydrophilic nature, these films are less effective as moisture barriers compared to synthetic alternatives. Furthermore, in order to reduce brittleness and prevent cracks in the matrix, hydrophilic plasticizers are often incorporated into protein films, mainly glycerol or sorbitol, which enhance the hydrophilicity of the resulting material [11,12]. An effective strategy to improve moisture resistance could involve exploiting the amphipathic properties of proteins. Lipids, with their ability to disperse within the protein matrix, can form either homogeneous emulgel films or bilayer films due to phenomena such as creaming or sedimentation of the lipid phase [13]. Unfortunately, the addition of lipids typically introduces microdomains within the protein film, which can disrupt protein–protein interactions, thereby weakening the material’s cohesive structural integrity. The properties of lipid-supplemented protein-based films are influenced by factors such as the polarity and degree of saturation of the added lipids, their concentration, placement within the polymer matrix, and lipid-protein interactions [14].

Candelilla wax (CW) is derived from the stems of the *Euphorbia antisyphilitica* shrub, native to desert regions in northern Mexico and the southwestern United States. It is a complex mixture containing wax hydrocarbons, resin esters, lactones, and free wax resin alcohols and acids. CW is yellow in color, brittle in texture, and melts between 56.84 and 79 °C. In the EU, CW is authorized as a glazing agent under the E 902 code [15,16]. Widely used in cosmetics, food coatings, and pharmaceuticals, it provides a firm texture, glossy finish, and moisture barrier. According to some studies [17], its water vapor permeability (WVP) is 7.4, 8.2, and ~74 times lower than that of beeswax, carnauba wax, and milk fat, respectively. Interestingly, CW also has significantly lower WVP than films made from PVDC, LDPE, polyester, and PVC (its permeability is 1.6, 2.6, 14, and 51 times lower, respectively). These characteristics suggest that CW could be highly effective as a moisture barrier [18], which is crucial for enhancing the stability and shelf life of foods sensitive to moisture transfer. Given its demonstrated advantages in terms of water vapor resistance, CW was considered an appropriate hydrophobing material for the films in our study. Additionally, its natural origin and widespread use in food-related applications further support its selection as a safe and effective choice.

However, it should be noted that the type of biopolymer and the processing conditions appear to be the most critical factors influencing the performance of the films. For example, bees wax was more effective than CW and carnauba wax in increasing the hydrophobicity of starch/gelatin edible films made by extrusion blowing [19]. In contrast, other studies have shown that CW and carnauba wax similarly reduced the WVP of sodium caseinate film [20].

Oleic acid (OA) is a monounsaturated omega-9 fatty acid commonly found in various plant and animal fats, especially olive oil, which contains 55–80% OA. As a result, hydrolysis of olive oil is a common way to obtain OA. With a melting point of approximately 13–16 °C, OA remains liquid at room temperature. Since OA contains only one carbon–carbon double bond, it exhibits stronger hydrophobicity and greater stability compared to polyunsaturated fatty acids [21,22]. Many studies have shown that the addition of OA can significantly reduce moisture uptake and/or transmission in biopolymer-based films [23,24]. However, its hydrophobic effect is generally weaker compared to natural waxes [18,25] or saturated fatty acids [26]. In addition to its functional properties, OA offers several health benefits, making it an attractive ingredient for edible films. As a key component of the Mediterranean diet, OA is widely recognized for its health benefits, including the prevention or slowing of cardiovascular diseases and cancer through mechanisms such as hypotensive effects, reduced glucose and insulin levels, improved cholesterol/HDL cholesterol ratios, and increased HDL cholesterol levels [27,28]. The inclusion of OA in films could provide functional advantages, such as improving water affinity, while also aligning with the growing demand for healthier, plant-based food packaging solutions.

The objective of the present study was to investigate how the concentration of CW and OA separately affects the structure, optical, water affinity, mechanical, and heat-sealing properties of PPI films plasticized with glycerol. We hypothesized that incorporating an appropriate amount of each lipid individually into the pea protein matrix could result in films with reduced WVP and/or improved water resistance, while simultaneously minimizing the negative impact on other properties, such as transparency and mechanical strength.

## 2. Results and Discussion

### 2.1. pH, Microstructure, and Viscosity of Film-Forming Solutions (FFSs)

**Figure 1:**
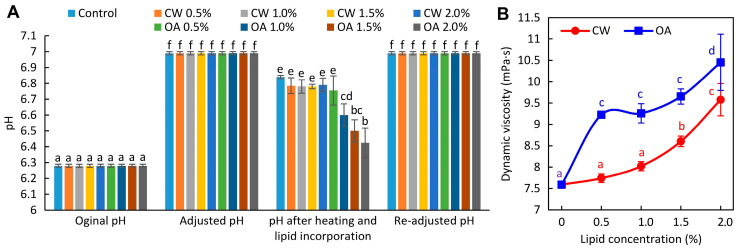
pH (**A**) and viscosity (**B**) of pea protein isolate-based film-forming solutions containing increasing concentrations of candelilla wax (CW) and oleic acid (OA). Values with different superscript letters (a–f) are significantly different (*p* < 0.05). The control refers to the lipid-free FFS.

Originally, before neutralization, the pH of the control FFS (mixture containing PPI, water, and glycerol) was 6.28 (Figure 1A). After heating and cooling, the pH of neutralized control FFS slightly decreased (to 6.84), consistent with our earlier observations [8]. As previously explained, this drop was likely due to the exposure of hidden carboxyl and amine groups, which may have altered the ionic balance and, consequently, the pH. The addition of CW had no impact on the pH of the FFS. Apparently, due to its highly non-polar nature, components of CW does not dissociate to release H⁺ ions. In turn, a significant reduction in pH (to 6.43–6.60), was observed after the addition of higher amounts of OA (≥1.0%, Figure 1A) likely due to the dissociation of OA’s carboxyl groups, leading to an increase in H⁺ concentration. Therefore, re-adjusting the pH of the FFSs was warranted to avoid values closer to the isoelectric point, which would be unfavorable for the film formation.

**Figure 2:**
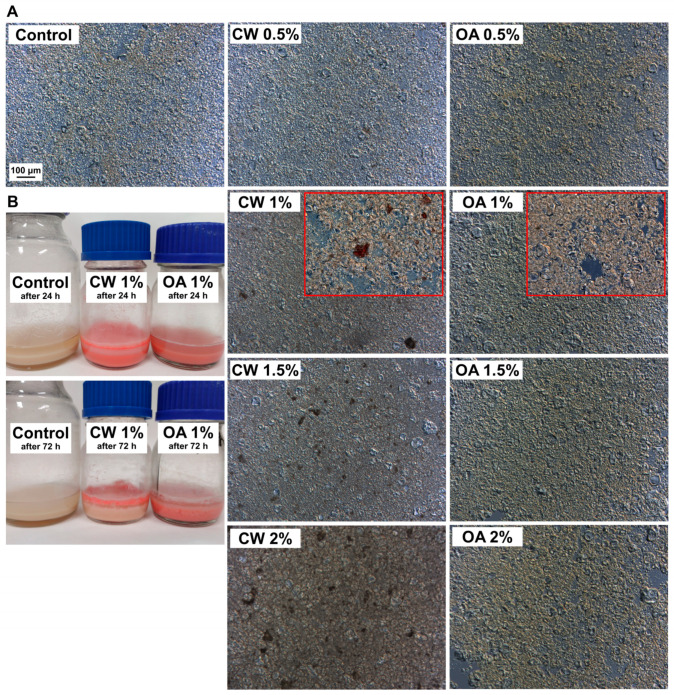
Effect of increasing concentrations of candelilla wax (CW) and oleic acid (OA) on the microtopography of pea protein isolate-based film-forming solutions (FFSs), visualized by differential interference contrast microscopy at 100× magnification. Red frames show 200× magnifications of FFSs obtained from lipids stained with Sudan Red III (**A**). Appearance of FFSs after storage at 25 °C (**B**). The control refers to the lipid-free FFS.

Since the pH of all cast FFSs was neutral (Figure 1A), the PPI did not fully dissolve and appeared as spherical particles with diameters reaching up to ~70 μm (Figure 2A), which is consistent with previous observations [18]. CW was observed in the emulsions as typically irregularly shaped particles, ranging in size from a few micrometers to ~70 μm. No OA droplets were detected in the emulsions, even after they were stained (Figure 2A), suggesting that OA was likely absorbed by the PPI. Thus, it can be concluded that OA exhibited a higher affinity for PPI than CW, which could favor the formation of OA–protein complexes. Studies of functional properties of proteins conducted by Webb et al. [29] showed that PPI can absorb sunflower oil in amounts ranging from approximately 0.5 to 1.0 g/g, depending on the manufacturing origin. So, the amount of PPI in the FFS was sufficient to ensure full absorption of OA. The comparison of microscopic images of FFSs containing CW and OA suggests that the latter were more translucent to light (Figure 2A), which is due to the fact that OA is a clear and colorless substance. Storage studies of FFSs obtained revealed PPI sedimentation (Figure 2B). CW underwent significant creaming. Such a phenomenon was not observed in OA-containing emulsions, again indicating that the PPI particles may have absorbed the liquid lipid.

Generally, a higher lipid addition resulted in greater resistance to flow in the FFS, as can be observed in Figure 1B. OA more efficiently thickened the FFS compared to the CW (9.23–10.45 vs. 7.74–9.58 mPa·s), which is consistent with previous study [18]. This can be explained in part by the fact that OA is a relatively viscous substance (29.31 mPa·s at 25 °C) [30]. It should also be noted that, although the particle size increase is less pronounced (Figure 2A and Figure S1) than in previous work [18], the larger size of OA-swelled PPI could lead to a greater increase in viscosity compared to CW, which existed as crystals in the aqueous phase and contributed less to viscosity.

### 2.2. Microstructure and pH of the Films

**Figure 3:**
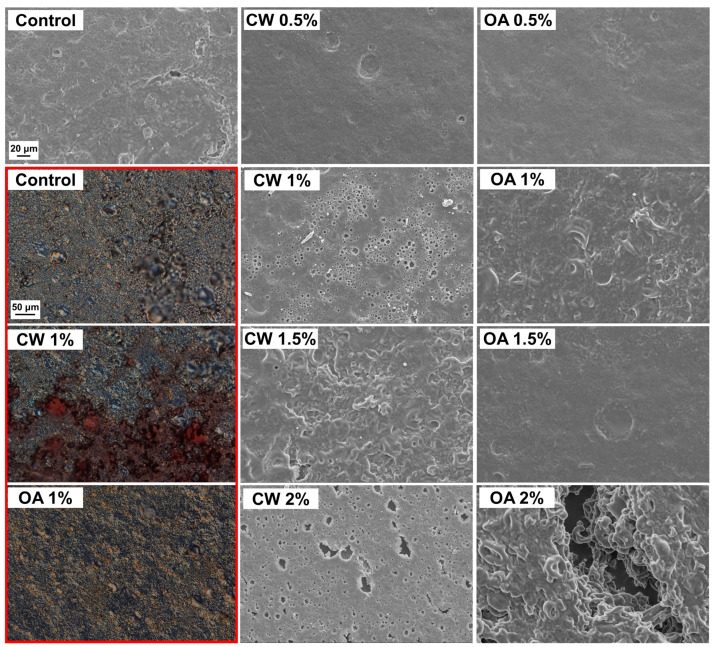
Microtopography of the air side of glycerol-plasticized pea protein isolate-based films without (control) and with increasing additions of candelilla wax (CW) and oleic acid (OA), visualized by scanning electron microscopy (grayscale images) and differential interference contrast (DIC) microscopy (images in red frame) at magnifications of 1000× and 200×, respectively. Emulsion films for DIC microscopy were prepared using lipids stained with Sudan Red III.

The obtained films comprised clusters of PPI particles likely bonded by the dissolved portion of the protein (Figure 3), which aligns with previous observations [8,18,31]. The porous surface structure observed in some microimages of the air side of films containing CW is most likely the separated lipid fraction, possibly including wax-protein complexes. Regions with varying degrees of this layer’s presence were detected. Numerous pores may result from water evaporation from the emulsion during film formation. However, these pores may also be markers of air microbubbles that diffused to the surface. It should be noted that an observation of the film’s bottom side microtopography (Figure S1) suggests that the wax was also present in the lower layer, as indicated by the irregular shape of the PPI particles, likely coated by CW.

**Figure 4:**
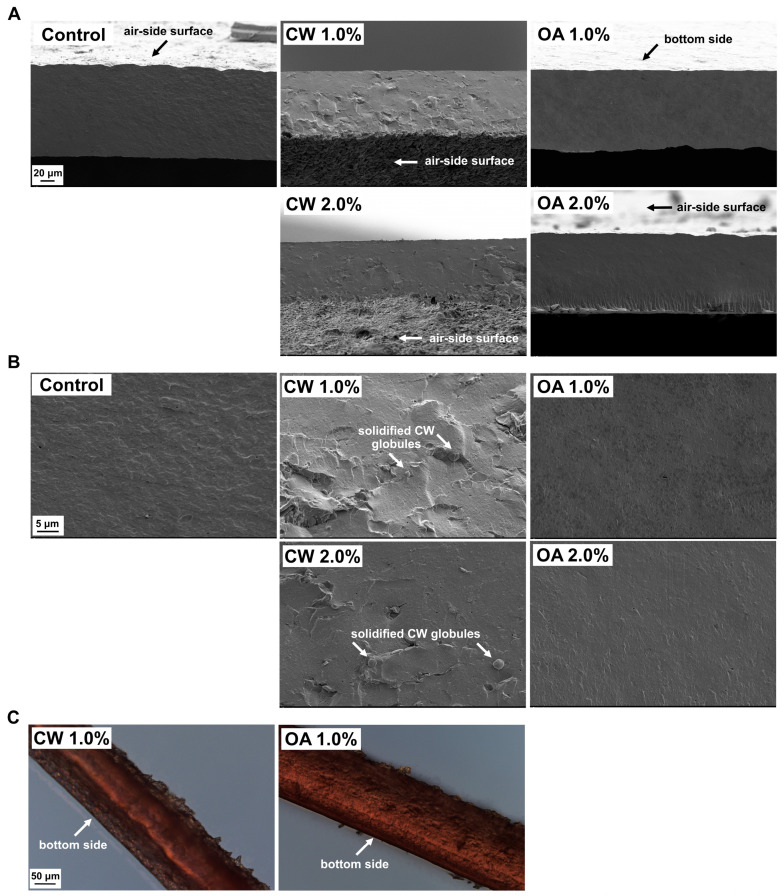
Cross-section of the glycerol-plasticized pea protein isolate-based films, without (control) and with additions of candelilla wax (CW) and oleic acid (OA), visualized by scanning electron microscopy at magnifications of (**A**) 1000× and (**B**) 5000×, as well as the films prepared with lipids stained with Sudan Red III, visualized by differential interference contrast (DIC) microscopy at a magnification of 200× (**C**).

All the obtained films had a compact cross-sectional structure (Figure 4). The most irregular fractures were observed in the CW-added films, which can be explained by the brittleness of the wax. In the SEM fractures of the CW-added films, it was possible to visualize globules of crystallized wax (Figure 4B). Though the SEM cross-section microimages of the films did not reveal phase separation (Figure 4A,B), the DIC images showed a three-layer structure in the CW-containing film (Figure 4C), which is consistent with the microtopography observations of both sides of the film (Figure 3 and Figure S1). Interestingly, the separated bottom layer was thicker than the layer on the air side.

Due to the discontinuities caused by 2.0% OA in the PPI film (Figure 3), no additional physicochemical testing was performed, although FTIR analysis was conducted to better understand the cause of this phenomenon. The observed voids were not typical fractures with a classic “rock candy” appearance (Figure 3), but rather gaps formed during film formation as a result of locally occurring reductions in adhesion between OA-soaked PPI particles. The negative effect of excessive OA addition on the cohesiveness of biopolymer-based films, including those made from PPI, has been previously reported [18,32,33]. It should be noted that for PPI films obtained from FFS containing 1.5% OA, edge cracking was observed; however, this did not prevent the acquisition of samples suitable for further testing.

The pH of the films was lower (Table 1) than that of the solutions from which they were cast (Figure 1A). This can be explained by the fact that as water evaporates, the amount of solvent decreases, leading to an increase in the concentration of dissolved substances, including ions responsible for acidity (H⁺). It was observed that only the pH of films with higher OA content (1.0–1.5%) differed significantly (*p* < 0.05) from the control film (6.58–6.62 vs. 6.76). As previously mentioned in Section 2.1, this was likely attributed to the dissociation of carboxyl groups in OA. Interestingly, the films containing CW and OA did not differ significantly in pH (Table 1).

### 2.3. ATR/FT-IR Analysis of the Films

**Figure 5:**
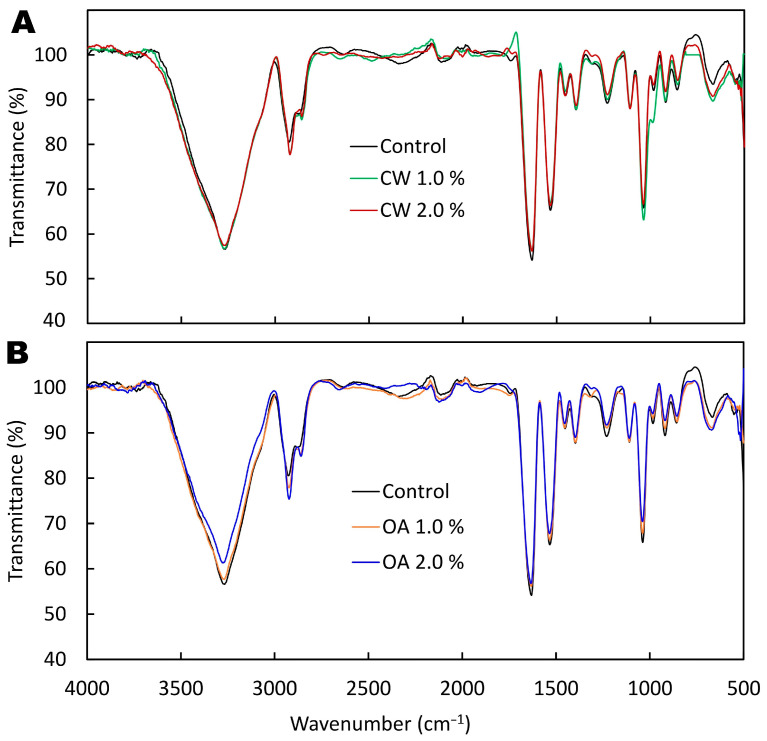
ATR/FT-IR spectra of the air side of glycerol-plasticized pea protein isolate-based films without (control) and with increasing additions of (**A**) candelilla wax (CW) and (**B**) oleic acid (OA).

The ATR/FT-IR spectra showed no new peaks or disappearance of any peaks after the addition of lipids to the PPI film (Figure 5). For example, the characteristic lipid signal in the 1740–1720 cm^−1^ range, typically associated with the C=O stretching (ν) vibrations of the carboxyl group in OA and the ester group (R-COO-R′) in CW [34,35,36,37], was not observed. Furthermore, no peak originating from the rocking deformation (ρ) vibrations of CH_2_ groups in long aliphatic hydrocarbon chains, in the ~715–725 cm^−1^ region [37,38] was detected either. This suggests that the existing functional groups/bonds in the main film components (PPI and glycerol) shielded the lipid signals. Nevertheless, differences in the intensity of certain bands were observed between the control and lipid-added films (Figure 5).

The spectra of all samples exhibited a broad peak at approximately 3270 cm^−1^**,** attributed to amide A stretching (Figure 5). Since this band does not appear in the spectra of pure CW and OA [34,36], its origin can be attributed to the –OH and –NH_2_ groups of the protein, as well as the –OH group from glycerol and water [39]. Interestingly, a reduced intensity of the amide A band was observed in the spectrum of 2% OA-added PPI film but not in the CW-added film. This suggests that OA may have a more significant effect on the self-interaction of protein functional groups or on the glycerol–protein interaction, e.g., hydrogen bond formation. As is known, the Amide A band depends on the strength of the hydrogen bonding of the NH group [37]. It is also possible that the irregular distribution of the wax layer on the film surface (Figure 3) prevented the detection of this change in the CW-added counterpart.

In contrast to the control, the emulsion-based films displayed slightly higher peaks at around 2921 cm^−1^ and 2858 cm^−1^, which can be attributed to the presence of long hydrocarbon chains in the both lipids, giving intense ν(C-H) vibrations from the methylene (CH_2_) groups and methyl groups (CH_3_) [37].

Consistent with the literature [40,41], characteristic peaks corresponding to amide I (~1631 cm^−1^), amide II (~1533 cm^−1^), and amide III (~1229 cm^−1^) were observed in spectra of all PPI-based films. These are primarily associated with the peptide bond (–CONH–), specifically: (i) the first corresponds to ν(C=O) vibration; (ii) the second mainly originates from NH bending vibrations in the plane of the bond (δ) and ν (CN) stretching, with a minor contribution from δ(C-O), ν(CC) or ν(NC); and the third is the result of the coupling of δ(NH) and ν(CN) vibrations with a small contribution from δ(C-O) and ν(C-C) [37]. The intensity of these peaks slightly decreased after the incorporation of lipids (Figure 5). This can likely be explained by the fact that the addition of lipids reduced the proportion of protein in the final FFS used to produce the films. Specifically, the same mass of non-aqueous components was cast, and as the lipid content increased, there was less protein and plasticizer in the FFS portion. The lipids may have also influenced pea protein conformation, potentially converting alpha-helices into a more random structure or reducing the amount of beta-turns [42]. These structural changes can be observed in the FTIR spectrum, where a decrease in the intensity of the amide I and II bands or shifts in their positions might indicate alterations in the protein’s secondary structure [37,43]. However, it is also possible that the lipids simply partially masked the protein’s characteristic bands.

Since glycerol was used as a plasticizer in the films, the two peaks around 1108–1038 cm^−1^ are most likely primarily attributed to the stretching vibrations of the C-O bonds in its hydroxyl groups [44]. Furthermore, the three small peaks in the ~855–983 cm^−1^ range were probably related to C-C skeletal vibrations in glycerol [45]. These bands were less intense after the addition of lipids, which may be related to the changes in the proportions of the components deposited on the casting plate. Conversely, the lipids induced an increase in the peak around 668 cm^−1^, likely due to more intense out-of-plane bending (γ) vibrations of the C-H bond [46] from alkyl chains.

### 2.4. Optical Properties of the Films

The addition of lipids, in most cases, caused darkening (a decrease in L* and whiteness index), yellowing, and a reduction in the intensity of the red color in the films. Despite these changes, the color difference (ΔE*) values for all obtained emulsion-based films were below 3.5 (Table 1), which means that a clear difference in color, compared to the control film, was not noticeable [47]. Films containing CW were more yellow than those with OA, which was most likely due to the yellow color of the wax. Increasing concentrations of CW and OA did not have a significant effect on the color parameters of the films (Table 1), which is consistent with previous results on sorbitol-plasticized PPI films [18].

**Figure 6:**
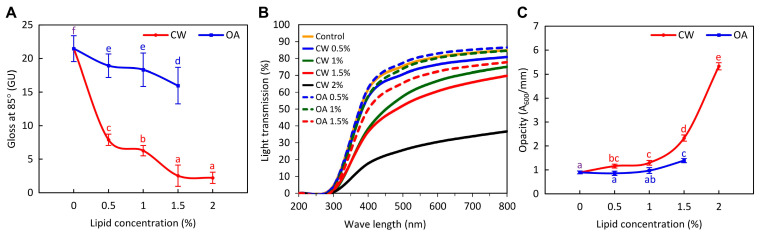
Effect of candelilla wax (CW) and oleic acid (OA) concentrations on the gloss (**A**), light transmittance (**B**), and opacity (**C**) of glycerol-plasticized pea protein isolate films. The control refers to the lipid-free film. Values with different superscript letters (a–f) are significantly different (*p* < 0.05).

Three basic angles of incidence, 60°, 20°, and 85°, are used for specular gloss measurement of material surfaces. According to EN ISO 2813 [48], the angle for gloss measurement should be selected based on the anticipated gloss range. Since the gloss of the air-exposed surface of all films at 60° was <10 GU (Figure S2), meaning they were matte, the 85° geometry was more suitable for differentiating between low-gloss films. At this angle, all emulsion films had a lower gloss surface compared to the control (Figure 6A). Increasing lipid content resulted in even more matte surfaces, presumably because the lipids disrupted uniform light reflection. Films containing CW were significantly less reflective than those with OA. Similarly, Fabra et al. [49] observed that OA reduced the gloss of sodium caseinate-based films to a lesser extent than beeswax or saturated fatty acids. The CW layer, which resulted in a high number of microdefects and irregularities on the film surface (Figure 3), likely caused intense light scattering, leading to a lower gloss appearance. It has been proved that an increase in surface roughness leads to a decrease in instrumentally measured surface gloss. It is worth noting, however, that this rule does not apply to visual perception of gloss [50]. As can be seen from the comparison of Figure S2 and Figure 6A, the angle of illumination also significantly affects the gloss data.

All the films completely blocked UVC light (<280 nm) and demonstrated excellent UVB light (280–315 nm) barrier properties, with light transmission (LT) ranging from 2% to 10% (Figure 6B), consistent with previous studies [8,18,31]. As it is known, the distinctive UV light absorption properties of proteins are attributed to chromophores in the side chains of aromatic amino acids: tryptophan, tyrosine, and phenylalanine (absorption maximum of approximately 280, 275, and 257 nm, respectively), as well as disulfide bonds (which exhibit weak absorption in the 250–320 nm range) and peptide bonds (showing strong absorption of approximately 190 nm and weaker absorption between 210 and 220 nm) [8,51,52]. The incorporation of CW decreased LT and, consequently, transparency in a concentration-dependent manner (Figure 6B,C). This result was due to the absorption and/or scattering of light by solidified wax particles dispersed throughout the films (Figure 4) [18,53]. OA did not reduce the transparency of the films up to a concentration of 1.0% (Figure 4C). Above this concentration, a significant decrease in LT was observed, consistent with previous study [54]. Some researchers suggest that this effect may result from light scattering at the interfaces of oil droplets embedded in the film matrix [55,56]. However, in our study, observations of both the FFS and the films did not reveal the presence of such droplets (Figure 2, Figure 3 and Figure 4), indicating that OA was effectively absorbed by the protein.

### 2.5. Water Affinities of the Films

In most cases, both CW and OA, regardless of concentration, equally reduced the moisture content (MC) of the PPI film (Table 2). Soaking tests showed that lipids were ineffective in limiting water molecules’ access to the protein matrix. After 1 h of shaking, both control and lipid-containing films disintegrated into several mushy pieces, making it difficult to conduct a precise analysis and requiring delicate handling of the water-soaked samples. Generally, in contrast to CW, the addition of OA significantly increased the film’s swelling (Sw) (Table 2). This suggests that OA might have acted as a more effective “disruptor” of protein–protein bonding, which likely led to increased porosity and/or a more loosely packed matrix structure, holding more water. A partial proof of this hypothesis is the idiopathically damaged structure of the film obtained from the FFS containing 2.0% OA (Figure 3) and significant changes in the FTIR spectrum, including the Amid A band (Figure 5).

Contrary to expectations, the addition of lipids also did not decrease the solubility (So) of the films (Table 2). On the contrary, both OA and CW, at the highest concentrations, increased the films’ susceptibility to disintegration (*p* < 0.5) (Table 2). This indicates that the lipids disrupted the protein network, probably reducing hydrogen bonds and hydrophobic interactions between protein chains, which likely made the film structure less compact and more prone to water interaction. This finding is consistent with Fakhouri et al. [57], who observed that higher levels of palmitic and caprylic fatty acids increased (by a few percent) the So of gelatin/gluten-based blend films. It is worth mentioning that the authors also demonstrated that other saturated fatty acids did not affect the films’ So. Also Gontard et al. [58] observed that beyond a certain lipid content, the So of emulsion-based gluten films sharply increased. Some authors, however, have observed improved water resistance (e.g., decreased solubility) of various protein-based films after the incorporation of lipids, including OA [56], attributing this phenomenon to the introduction of a hydrophobic phase, which is not accessible to water [59]. It should also be mentioned that the observed differing effects of lipids on So results may, in part, be due to different methodological approaches. Some authors use for testing heat-dried (~100 °C, 24 h) films (residue after oven drying for MC determination) instead of acclimated samples, and they express So as the amount of undissolved dry mass after 24 h. As shown, the dehydro-thermo-treatment of films develops tensional and compressional stresses in the material, which improves its integrity throughout the soaking procedure [60]. Therefore, the method used in this study accurately reflects the behavior of PPI films after coming into 1 h contact with water.

Except for the lowest level of OA addition (0.5%), lipid incorporation significantly improved the water vapor barrier properties, generally in a concentration-dependent manner (Table 2). The CW, which migrated to the surfaces (Figure 3 and Figure 4), provided a greater reduction in WVP (approximately 37–63%, depending on the concentration) compared to the OA, which was absorbed by PPI and reduced WVP by only 2–18%. It is well known that bilayer films, including those formed due to destabilization-induced lipid concentration gradients, are more effective in preventing moisture transfer than emulgels, where moisture can penetrate between the dispersed lipid phase [59]. Furthermore, as mentioned in the introduction section, among various natural and synthetic materials, CW has the lowest WVP [17], likely due to its high hydrocarbon content [61]. As a result, the largely separated CW, in contrast to OA (Figure 3 and Figure 4), increased the contact angle (CA), i.e., the hydrophobicity of the PPI film surface, in a concentration-dependent manner (Table 2).

### 2.6. Mechanical Properties of the Films

Unlike OA, even a small addition of CW (0.5%) reduced both tensile strength (σ_max_) and elastic modulus (EM) of the PPI film (Table 3), indicating that the crystals and globules (Figure 3 and Figure 4) weakened the structural integrity of the proteinaceous matrix. Although a small addition of OA did not reduce the parameters mentioned above, the significant decrease in elongation at break (ε_b_) (Table 3) also suggests a reduction in adhesion between pea protein particles. In general, a higher lipid addition led to a greater deterioration in the mechanical parameters, though this was not always the case. Overall, the addition of lipids decreased σ_max_ values by approximately 28–37% and EM by approximately 18–43% (Table 3). At higher incorporation levels (1.0–1.5%), the presence of lipids resulted in a similar weakening of the film’s strength and stretchability.

### 2.7. Hot Seal Strength (HSS) of the Films

Heat sealability is a key property of packaging materials, as it allows for the formation of hermetic seals that protect the product from moisture, air, and contaminants, extending shelf life and ensuring safety. It was found that too high sealing jaw temperatures (>90 ± 10 °C, with a sealing time of 2 s) resulted in excessively fused seams, suggesting possible thermal degradation of the film components. Additionally, in the CW-containing films, excessive melting reduced the visual quality of the film around the seal. As reported in the literature [62], higher temperatures also resulted in bubble formation within the sealing area. The heat sealability of PPI-based film was improved by adding CW and OA, but only at levels of 1.0% and 0.5%, respectively. With these additions, HSS increased by 42% and 52%, respectively (Table 3). In the CW-added films, the enhanced bonding appeared to result from interlayer wax melting. In turn, OA-enhanced adhesion was likely due to its plasticizing effect, allowing for better material moulding and distribution under pressure. The plasticizing effect of OA is well-documented [63], and some sources suggesting that plasticizers are essential to impart heat-sealing ability. For example, this situation is evident in materials like starch [64], which lacks thermoplastic properties due to its semi-crystalline structure and strong intermolecular bonds that resist heat-induced transformation. It should be noted that CW and OA, in amounts greater than optimal, did not significantly impact HSS, suggesting that the weakened mechanical structure of the films (Table 3) does not favor improvement in heat sealability of the PPI film.

Research on the heat-sealability of various biopolymer-based films, including those made from proteins, is growing rapidly. Comparing results is challenging, as seal quality depends not only on temperature and sealing time but also on pressure, as well as the amount and type of plasticizer [62,64]. The HSS observed in this study (0.069–0.105 N/mm, Table 3) is similar to that reported for glycerol-plasticized soy protein isolate (SPI)-based film (0.076 N/mm) [65]. In Lu et al. study [62], the HSS of glycerol-plasticized SPI-based films enriched with diatomite/thymol complex reached a peak of 0.16 N/mm at 140 °C (within the 110–150 °C range), which, according to the authors, was primarily due to heat-induced disulfide cross-linking. In turn, Tai et al. [66] showed that glycerol-plasticized SPI-based films could be effectively sealed at temperatures between 180 and 230 °C, as seal strength improved with increasing temperature. Notably, these authors demonstrated that increasing glycerol content (from 1% to 3%) reduced HSS (from ~0.4 to ~0.2 N/mm), as higher plasticizer levels seemed to diminish cohesion between protein chains, thereby weakening the seal. Kim and Ustunol [67] reported a maximum HSS of approximately 0.3 N/mm at 110 and 130 °C for glycerol- and sorbitol-plasticized whey protein isolate (WPI)-based films, respectively, which aligned with the films’ onset temperatures. Seal formation in these films was driven mainly by hydrogen and covalent bonding, such as C–O–H and N–C bonds. The authors also noted mixed effects of CW on HSS, with variations depending on sealing conditions and the type of plasticizer used. Janjarasskul et al. [68] observed HSS of up to ~0.4 N/mm in WPI films, especially those derived from thermally denatured proteins. Gelatin films are known for their good heat-sealing properties. Fish gelatin films can be sealed at temperatures near or above their melting transition (150 °C) with a sealing time 1–1.5 s [69]. Control fish gelatin film showed higher HSS compared to emulsion films containing oils or surfactants (~0.57–0.78 vs. ~0.03–0.50 N/mm, depending on heating time). According to the authors, this difference was just attributed to the higher σ_max_ and EM values of the control films. In another study [70], the maximum HSS of gelatin films approached 1 N/mm. The addition of ZnO nanorods significantly increased HSS, likely due to the formation of hydrogen and other bonds, but excessive ZnO reduced HSS, probably due to decreased moisture content and, thus, reduced flexibility.

In summary, the PPI-based films obtained in this study offered relatively low HSS when compared to WPI- and gelatin-based films, and their heat sealability was similar only to those reported in one study on soy protein-based films.

## 3. Materials and Methods

### 3.1. Materials

Propulse PPI (with a protein content of 82 ± 2%) was kindly provided by Nutri-Pea (Portage la Prairie, MB, Canada). Glycerol (min. 99.5%), CW, and OA (90%) were obtained from Sigma Chemical Co. (St. Louis, MO, USA).

### 3.2. Methods

#### 3.2.1. Preparation of the Film-Forming Solutions (FFSs) and Films

The films were produced by evaporating FFSs composed of 10% (*w*/*w*) PPI, 4% (*w*/*w*) glycerol, and varying concentrations of CW or OA (0%, 0.5%, 1.0%, 1.5%, and 2.0% *w*/*w*). Initially, the PPI, glycerol, and distilled water mixture was neutralized using concentrated NaOH solution to achieve a pH of 7.0 ± 0.05, and heated in a water bath at 90 °C for 20 min with continuous stirring. Prior to the end of heating, the lipid was incorporated, and the solution was then mixed using a homogenizer (H-500, Pol-Eko Aparatura, Wodzisław Śląski, Poland) at 20,000 rpm for 5 min. The FFSs were subsequently cooled to 25 °C with continuous stirring, after which their pH was adjusted back to 7.0. Following this, the FFSs were re-homogenized at 20,000 rpm for 1 min, degassed, and poured into polystyrene Petri dishes (145 cm^2^ area; Nunc, Roskilde, Denmark). To ensure a uniform film thickness of 100 ± 10 μm, 1.5 g of total solids (PPI + glycerol + lipid) was cast onto the leveled Petri dish, equating to approximately 9.4–10.7 g of FFS, depending on the lipid concentration. The films were dried for approximately 24 h at ~25 °C and 50% ± 5% RH. Afterward, the films were cut into samples, and their thickness was measured using a Mitutoyo No. 7327 micrometer (Mitutoyo, Tokyo, Japan). The samples were conditioned at 25 °C and 50% RH for 48 h in a test chamber (MLR-350H, Sanyo Electric Biomedical Co., Ltd., Oizumi-Machi, Japan).

#### 3.2.2. Characterization of the FFSs

A glass electrode (Elmetron ERH-11S, Zabrze, Poland) linked to a pH meter (Elmetron CPC 401, Zabrze, Poland) was employed to monitor the pH of the FFSs at 25 ± 1 °C throughout various stages of their preparation.

The FFSs’ microstructure was analyzed using a Leica 5500B microscope (Leica Microsystems GmbH, Wetzlar, Germany) equipped with a differential interference contrast (DIC) optical system. Additionally, the FFSs containing lipids stained with Sudan Red III were examined under the microscope and stored at room temperature for 72 h to observe their behavior, specifically any morphological instability.

The dynamic viscosity of the FFSs was assessed using a rotational viscometer (ROTAVISC lo-vi, IKA, Staufen, Germany) with the VOLS-1 adapter and spindle VOL-SP-6.7, operating at 200 rpm and 23 °C, using 6.7 mL of the sample. All measurements were carried out in triplicate.

#### 3.2.3. Microstructure of the Films

The microtopography of the films, including both the air-exposed surface (during drying) and the cross-section (after immersion in liquid nitrogen and fracturing), was examined using a scanning electron microscope (Zeiss Ultra Plus, Oberkochen, Germany). Prior to observation, the samples were re-dried under vacuum and coated with a 15 nm gold layer. Additionally, films prepared with lipids stained using Sudan Red III were analyzed using DIC microscopy.

#### 3.2.4. Attenuated Total Reflectance Fourier Transform Infrared Spectroscopy (ATR/FT-IR) Study of the Films

The FT-IR spectra of the films were recorded with an Agilent Cary 630 FT-IR spectrometer (Agilent Technologies, Inc., Santa Clara, CA, USA) using the ATR technique. All spectra were obtained at room temperature after averaging 32 scans between 500 and 4000 cm^−1^ with a resolution of 4 cm^−1^ in the transmittance mode. The samples spectra were recorded using the Micro Lab FTIR software (version B.04). Additionally, the Agilent Resolutions Pro software (version 5.2.0.861) was used for the post-collect analysis of spectra.

#### 3.2.5. pH of the Films

A flat surface electrode (Elmetron EPX-3, Zabrze, Poland) linked to a pH meter (Elmetron CPC 401, Zabrze, Poland) was employed to measure the pH of the films after hydrating the surface with 20 μL of deionized water.

#### 3.2.6. Optical Properties

The color values (CIE L*a*b*) of the film samples (1 × 4 cm) were determined using an X-Rite Color 8200 colorimeter (X-Rite Inc., Grand Rapids, MI, USA) against a black background (L* = 25.63, a* = −0.12, b* = −0.47). The whiteness index (WI) was then calculated using Equation (1)

(1) $$WI=100-\sqrt{{\left(100-L^{*}\right)}^{2}+a^{*2}+b^{*2}}$$

The total color difference (ΔE*) was calculated using Equation (2)

(2) $$\Delta E^{*}=\sqrt{\Delta L^{*2}+\Delta a^{*2}+\Delta b^{*2}}$$

where Δ represents the difference between the color parameters of the film without lipids (control) and those containing the lipids.

The gloss of the air-exposed surface of the film was measured at 20°, 60°, and 85° angles using a NHG268 gloss meter (3nh, Guangzhou, China) against a black background.

The light-barrier properties of the film samples (1 × 4 cm) were assessed using a spectrophotometer (Lambda 40, Perkin–Elmer, Shelton, CT, USA) at selected wavelengths ranging from 200 to 800 nm. The spectrophotometer was first calibrated with air. The film sample was then placed in the designated slit, where a cuvette is normally positioned, and a UV/Vis spectrum scan was performed. The opacity of the films was calculated using Equation (3)

(3) $$\mathrm{Opacity}\;=\frac{A_{600}}{t}$$

where A_600_ is the absorbance of the film sample at 600 nm and t is the film sample thickness (mm).

The optical analyses were conducted at least five times.

#### 3.2.7. Water Affinities

The film specimens (2 × 2 cm) were dried in an oven at 105 °C for 24 h. The moisture content (MC) was calculated by determining the percentage of water lost from the samples. The samples of the same dimensions (2 × 2 cm), weighed to the nearest 0.001 g, were then shaken with 30 mL of distilled water in 50 mL Falcon test tubes in an ES-60 incubator (MIULAB, Hangzhou, China) at 25 ± 1 °C and 70 rpm for 1 h. Afterward, the films in their swollen state were gently blotted with tissue and re-weighed. The swelling (Sw) degree was expressed as the percentage increase in mass of the film. The remaining sample after the test was placed in a pre-weighed dish and conditioned at 25 °C and 50% RH for 48 h. Solubility (So) was calculated as the percentage of the film dissolved in water. All analyses of MC, Sw, and So were carried out in quadruplicate.

WVP (g mm m^−2^ day^−1^ kPa^−1^) was calculated as follows:

(4) $$\mathrm{WVP}\;=\frac{\mathrm{WVTR}\;\times \;t}{\Delta_{p}}$$

where WVTR is the water vapor transmission rate (g m^−2^ day^−1^) measured gravimetrically based on the ISO 2528 method [71], t is the mean film thickness (mm), and Δ_p_ is the difference in the water vapor pressure (kPa) between two sides of the film.

In brief, poly (methyl methacrylate) permeation cell cups with an internal diameter of 7.98 cm (exposed film area = 50 cm^2^) and an internal depth of 2 cm were filled with 10 g of anhydrous calcium chloride (0% RH). Film samples with a diameter of 10 cm were placed over the circular openings and sealed with O-ring rubber gaskets and screw tops. The cups were then placed in a test chamber at 25 °C and 50% RH. Weight gain was monitored over 12 h, with measurements taken every 2 h. The slopes of the steady-state (linear) sections of the weight gain versus time curves were used to calculate the WVTR. The WVP analyses were performed in triplicate.

A droplet of distilled water was carefully applied to the air side of the film using a microsyringe, while the droplet image was simultaneously captured using an Olympus SZX10 microscope (Olympus, Hamburg, Germany) equipped with a camera. The average contact angle (CA) was calculated using Ossila Contact Angle software 4.2.1 (Ossila Ltd., Sheffield, UK). The CA analysis was carried out in quadruplicate.

#### 3.2.8. Mechanical Properties

Tensile strength (σ_max_), elongation at break (ε_b_), and elastic modulus (EM) were measured in at least eight repetitions using a TA-XT2i texture analyzer fitted with a 50 kg load cell (Stable Micro Systems, Godalming, UK), following the procedure described in PN-EN ISO 527-1, 2, 3:1998, with some modifications [72]. The dumbbell-shaped film samples were mounted on the analyzer with an initial grip separation of 80 mm and stretched at a speed of 1 mm s^−1^. The aforementioned parameters were calculated using Equations (5)–(7), respectively:

(5) $$\sigma_{max}=\frac{F_{\max}}{A}$$

where F_max_ is the maximum load required to break the film (N), and A is the initial cross-sectional area (thickness × width, mm^2^) of the specimen.

(6) $$\varepsilon_{b}=\frac{\Delta L}{L_{i}}\times 100$$

where ΔL is the change in length at the moment of fracture, and L_i_ is the initial gage length (mm).

(7) $$\mathrm{EM}\;=\frac{\sigma_{2}-{\;\sigma }_{1}}{\varepsilon_{2}-{\;\varepsilon }_{1}}$$

where ε_1_ is a strain of 0.0025 (0.25%), ε_2_ is a strain of 0.01 (1%), σ_1_ (MPa) is the stress at ε_1_ and σ_2_ (MPa) is the stress at ε_2_.

#### 3.2.9. Heat-Sealing Properties

Heat sealing was carried out using an adjustable PFS-200 impulse sealer (Yongkang Golden Sky Imp. & Exp. Co., Ltd., Zhiying Town, China). The air sides of conditioned film strips (2 × 5 cm) were aligned parallel, sealed along the shorter side at program 4 (temperature: 90 ± 10 °C, time: 2 s) approximately 1 cm from the edge, and immediately removed from the sealer jaws. The device’s design ensured sealing under constant pressure. The temperature between the jaws was monitored using a K-type TP-01 thermocouple connected to a TM-902C thermometer (Shenzhen VSEC Electronic Co., Ltd., Guangzhou, China). The sealed films were then reconditioned at 25 °C and 50% RH for 48 h. Next, the film samples were mounted on the tensile grips of a TA-XT2i texture analyzer with an initial separation of 30 mm (so that the seal was at the midpoint between the grips) and stretched at a speed of 1.5 mm s^−1^. The maximum force required to peel the seal apart was recorded and divided by the seal length (20 mm) to obtain the hot seal strength (HSS) in N/mm. The measurements were performed in six repetitions.

#### 3.2.10. Statistical Analysis

Statistical differences among the mean values were evaluated for significance at the *p* < 0.05 level using analysis of variance (ANOVA) and Fisher’s test (STATISTICA 13.3, StatSoft Inc., Tulsa, OK, USA).

## 4. Conclusions

In contrast to OA, CW increased surface hydrophobicity, which was likely responsible for the greater reduction in WVP (37–63% vs. 2–18%, depending on the concentration). It indicates that CW-added PPI films are more promising candidates for packaging moisture-sensitive foods than the OA-containing films. Although OA had a smaller impact on optical properties, especially opacity, compared to CW, it caused a 20–30% increase in water absorption, a 13–18% increase in solubility, and inhibited cohesive film formation at the 2% concentration. Both CW and OA reduced the films’ mechanical strength (up to ~36%) and stretchability (even by as much as half) in a fairly similar manner. On the other hand, at optimal concentrations, both CW and OA significantly enhanced heat-sealing efficiency, with increases of 42% and 52%, respectively, making the PPI films more suitable for thermal welding applications.

The findings of this study highlight the importance of selecting and optimizing the concentration of hydrophobizing agents, such as CW and OA, to improve the functional properties of protein-based films. As potential future research directions, the use of a mixture of OA and CW for the hydrophobization of the PPI film seems to be an interesting avenue to explore.

## Figures and Tables

**Table 1:** Effect of candelilla wax (CW) and oleic acid (OA) concentrations on the color parameters (L*, a*, b*), whiteness index (WI), and color difference (ΔE*) (between control (lipid-free) and lipid-added samples) of glycerol-plasticized pea protein isolate films.

Lipid Type	Lipid Content (%)	pH	L*	a*	b*	WI	ΔE*
Control	0	6.76 ± 0.11 ^b^	41.39 ± 0.01 ^c^	−0.21 ± 0.03 ^c^	2.13 ± 0.07 ^a^	41.35 ± 0.01 ^c^	-
CW	0.5	6.69 ± 0.02 ^ab^	42.05 ± 0.48 ^c^	−076 ± 0.15 ^a^	5.21 ± 0.05 ^d^	41.81 ± 0.48 ^c^	3.22 ± 0.16 ^a^
	1.0	6.66 ± 0.03 ^ab^	39.98 ± 1.71 ^b^	−0.79 ± 0.03 ^a^	4.12 ± 0.37 ^c^	39.83 ± 1.70 ^b^	2.75 ± 1.08 ^a^
	1.5	6.65 ± 0.07 ^ab^	39.21 ± 0.30 ^ab^	−0.88 ± 0.08 ^a^	4.03 ± 0.33 ^c^	39.07 ± 0.39 ^ab^	2.96 ± 0.50 ^b^
	2	6.69 ± 0.08 ^ab^	39.51 ± 0.21 ^ab^	−0.69 ± 0.02 ^ab^	4.94 ± 0.49 ^d^	39.45 ± 0.27 ^ab^	2.87 ± 0.49 ^a^
OA	0.5	6.71 ± 0.06 ^ab^	39.94 ± 1.22 ^b^	−0.46 ± 0.02 ^b^	2.69 ±0.46 ^b^	39.87 ± 1.21 ^b^	1.67 ± 1.12 ^a^
	1.0	6.62 ± 0.02 ^a^	38.54 ± 0.86 ^a^	−0.65 ± 0.17 ^ab^	2.68 ± 0.44 ^ab^	38.47 ± 0.84 ^a^	2.97 ± 0.79 ^b^
	1.5	6.58 ± 0.15 ^a^	39.62 ± 0.412 ^ab^	−0.50 ± 0.05 ^b^	3.23 ± 0.18 ^b^	39.53 ± 0.16 ^ab^	2.11 ± 0.16 ^ab^

Values with different superscript letters (a–d) within a single column are significantly different (*p* < 0.05).

**Table 2:** Effect of candelilla wax (CW) and oleic acid (OA) concentrations on the moisture content (MC), swelling (Sw), solubility (So), water vapor permeability (WVP), and contact angle (CA) of glycerol-plasticized pea protein isolate films. The control refers to the lipid-free film.

Lipid Type	Lipid Content (%)	MC (%)	Sw (%)	So (%)	WVP (g mm m^−2^ day^−1^ kPa^−1^)	CA (°)
Control	0	22.73 ± 1.20 ^b^	203.28 ± 19.35 ^a^	47.22 ± 2.53 ^a^	14.30 ± 0.68 ^f^	41.22 ± 6.66 ^a^
CW	0.5	22.80 ± 0.32 ^b^	222.32 ± 4.73 ^abc^	50.33 ± 3.68 ^ab^	8.96 ± 0.32 ^c^	74.71 ± 7.41 ^b^
	1.0	19.99 ± 0.48 ^a^	238.36 ± 13.35 ^bcd^	51.73 ± 1.10 ^abc^	8.75 ± 0.51 ^c^	64.77 ± 4.01 ^b^
	1.5	20.62 ± 1.24 ^a^	223.33 ± 1.57 ^abc^	51.78 ± 3.43 ^abc^	6.89 ± 0.42 ^b^	97.60 ± 26.73 ^c^
	2	19.19 ± 0.73 ^a^	208.91 ± 15.40 ^ab^	57.40 ± 2.02 ^d^	5.36 ± 0.22 ^a^	100.13 ± 20.33 ^c^
OA	0.5	19.86 ± 1.33 ^a^	247.20 ± 23.72 ^cd^	53.26 ± 0.67 ^bcd^	13.96 ± 1.02 ^ef^	36.25 ± 6.82 ^a^
	1.0	20.06 ± 1.03 ^a^	245.17 ± 30.11 ^cd^	55.86 ± 4.77 ^cd^	12.90 ± 1.01 ^e^	28.95 ± 3.65 ^a^
	1.5	19.53 ± 0.95 ^a^	263.88 ± 31.14 ^d^	54.13 ± 4.24 ^bcd^	11.72 ± 0.68 ^d^	24.28 ± 2.08 ^a^

Values with different superscript letters (a–f) within a single column are significantly different (*p* < 0.05).

**Table 3:** Effect of candelilla wax (CW) and oleic acid (OA) concentrations on the tensile strength (σ_max_), elongation at break (ε_b_), elastic modulus (EM), and hot seal strength (HSS) of glycerol-plasticized pea protein isolate films. The control refers to the lipid-free film.

Lipid Type	Lipid Content (%)	σ_max_ (MPa)	ε_b_ (%)	EM (MPa)	HSS (N/mm)
Control	0	3.56 ± 0.28 ^c^	81.23 ± 7.97 ^e^	99.11 ± 5.81 ^d^	0.069 ± 0.016 ^ab^
CW	0.5	2.70 ± 0.20 ^b^	50.38 ± 11.45 ^c^	71.14 ± 5.26 ^bc^	0.060 ± 0.030 ^a^
	1.0	2.56 ± 0.25 ^ab^	55.44 ± 14.68 ^cd^	70.04 ± 3.60 ^b^	0.098 ± 0.015 ^cd^
	1.5	2.50 ± 0.27 ^ab^	39.88 ± 4.00 ^ab^	72.20 ± 9.32 ^bc^	0.063 ± 0.033 ^a^
	2	2.28 ± 0.13 ^a^	33.68 ± 5.01 ^a^	69.09 ± 6.12 ^b^	0.073 ± 0.006 ^abc^
OA	0.5	3.61 ± 0.40 ^c^	64.19 ± 10.28 ^d^	99.63 ± 16.44 ^d^	0.105 ± 0.022 ^d^
	1.0	2.57 ± 0.48 ^ab^	57.28 ± 7.60 ^cd^	81.47 ± 17.74 ^c^	0.091 ± 0.009 ^bcd^
	1.5	2.25 ± 0.36 ^a^	47.02 ± 5.44 ^bc^	56.76 ± 6.86 ^a^	0.088± 0.008 ^abcd^

Values with different superscript letters (a–e) within a single column are significantly different (*p* < 0.05).

## Data Availability

The raw data supporting the conclusions of this article will be made available by the authors on request.

## Supplementary Materials

The following supporting information can be downloaded at: https://www.mdpi.com/article/10.3390/molecules29245998/s1, Figure S1: Microtopography of the bottom side of glycerol-plasticized pea protein isolate-based films, obtained from film-forming solutions containing 2% candelilla wax (A) and oleic acid (B), visualized by scanning electron microscopy at 2500× magnification. Figure S2: Effect of candelilla wax (CW) and oleic acid (OA) concentrations on the gloss of glycerol-plasticized pea protein isolate films at 20° (A) and 60° (B).
