# Supplementary material for: Structural and Physicochemical Properties of Glycerol-Plasticized Edible Films Made from Pea Protein-Based Emulsions Containing Increasing Concentrations of Candelilla Wax or Oleic Acid
Source: Molecules. 2024 Dec 19;29(24):5998. doi: 10.3390/molecules29245998 (PMC11676376; doi:10.3390/molecules29245998)
Supplement: Supplementary file 1 [file molecules-29-05998-s001.zip › molecules-3344143-supplementary.pdf]

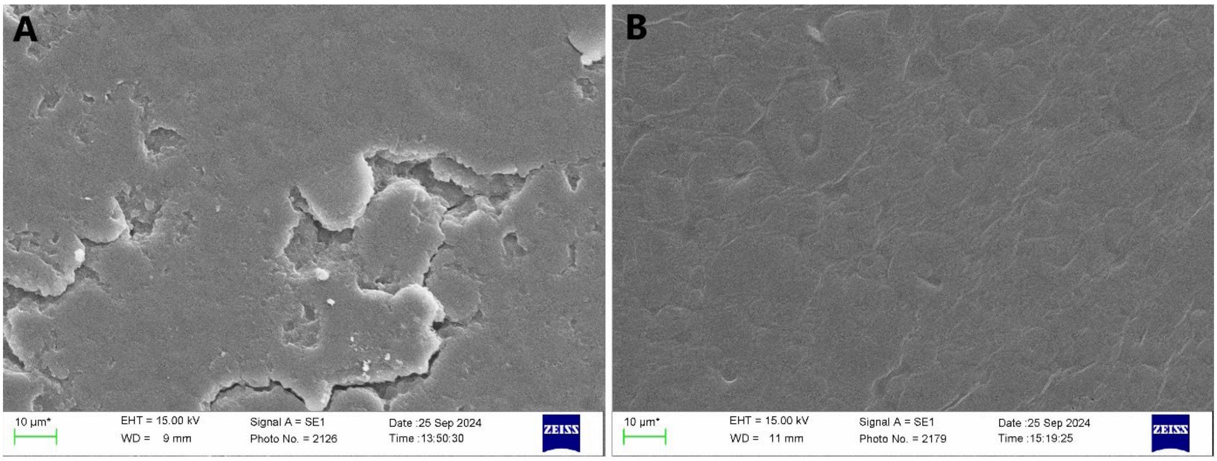

Figure S1. Microtopography of the bottom side of glycerol-plasticized pea protein isolate-based films, obtained from film-forming solutions containing 2% candelilla wax (**A**) and oleic acid (**B**), visualized by scanning electron microscopy at 2500× magnification.

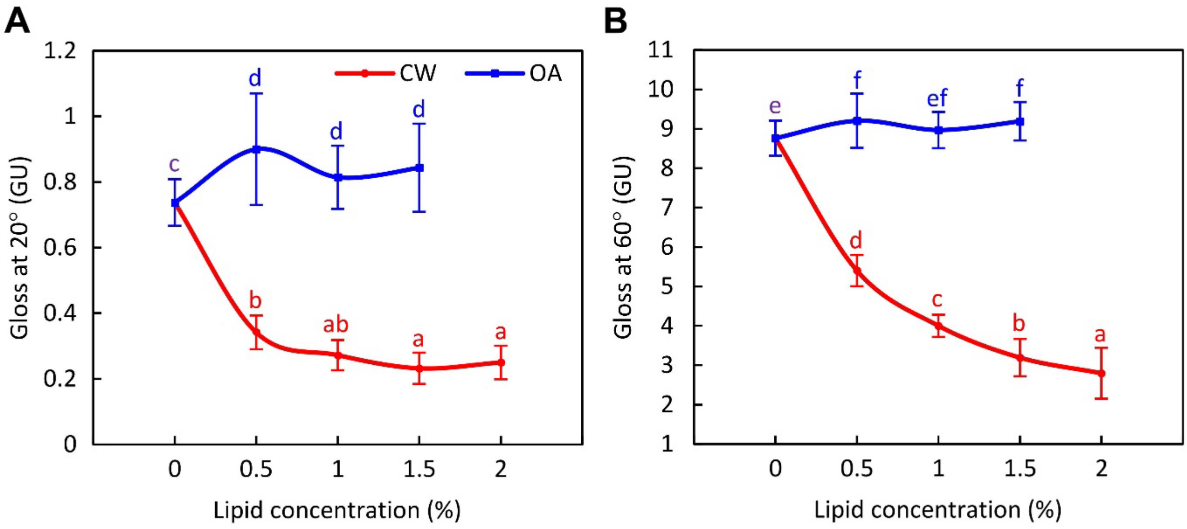

Figure S2. Effect of candelilla wax (CW) and oleic acid (OA) concentrations on the gloss of glycerol-plasticized pea protein isolate films at 20° (**A**) and 60° (**B**).
